# Maternal Electronic Cigarette Exposure Induces Dysregulation of Autophagy via Oxidative Stress/DNA Methylation in Pulmonary Hypertension Offspring

**DOI:** 10.1007/s11596-025-00074-8

**Published:** 2025-06-24

**Authors:** Ze-wen Chen, Yi-fan Li, Hai-long Qiu, Wen Xie, Tian-yu Chen, Yong Zhang, Ji-mei Chen, Jian Zhuang, Shu-sheng Wen

**Affiliations:** 1 Department of Cardiac Surgery, Guangdong Cardiovascular Institute, Guangdong Provincial People’s Hospital (Guangdong Academy of Medical Sciences), Southern Medical University, Guangzhou, 510080 China; 2 Department of Pediatric Cardiology, Guangdong Cardiovascular Institute, Guangdong Provincial People’s Hospital (Guangdong Academy of Medical Sciences), Southern Medical University, Guangzhou, 510080 China

**Keywords:** Electronic cigarette, Maternal, Pulmonary hypertension, Offspring, Oxidative stress, DNA methylation, Autophagy

## Abstract

**Objective:**

Electronic cigarettes (ECs) differ from traditional tobacco smoke but may contribute to cardiopulmonary remodeling. Pulmonary hypertension (PH), characterized by pulmonary artery and right ventricle remodeling, poses a significant risk of mortality in infants, children, and adolescents. However, the impact of maternal EC exposure on PH development in offspring remains unclear. To address this, we established a PH rat model with maternal EC exposure.

**Methods:**

Maternal EC exposure was initiated on gestation day 12 via electronic nicotine delivery systems. Offspring were administered monocrotaline (MCT) at 6 weeks of age (6-wo) to induce PH. Mechanistic experiments were conducted at 10-week-old (10-wo). Protein expression of NADPH oxidases, DNA methyltransferases, and autophagy-related markers was analyzed by Western blot. Morphological changes and the severity of PH were evaluated via hematoxylin and eosin (HE) staining and echocardiography, respectively. Furthermore, the involvement of the oxidative stress/DNA methylation/autophagy axis in response to maternal EC exposure was confirmed through a combination of ELISA, Western blot, HE staining, and echocardiography. Additionally, ATG5 mRNA expression was measured by qRT-PCR.

**Results:**

Compared with control conditions, maternal EC exposure significantly worsened MCT-induced PH in male offspring. This was associated with increased oxidative stress, DNA hypomethylation, and anomalous autophagy in the offspring. In vivo treatment with chloroquine inhibited autophagy and ameliorated PH development in offspring exposed to maternal EC. Furthermore, N-acetylcysteine (NAC), an antioxidant, attenuated maternal EC exposure-induced oxidative stress, DNA hypomethylation, and excessive autophagy, thereby improving PH. DNA hypermethylation also reversed PH development, accompanied by reduced oxidative stress and suppressed autophagy. ATG5, a key regulator of autophagy, was identified as a potential therapeutic target, as its repression mitigated PH in maternal EC-exposed offspring.

**Conclusion:**

Maternal EC exposure induces oxidative stress and DNA hypomethylation in offspring, leading to anomalous autophagy and exacerbation of PH development. Targeting ATG5-mediated autophagy may represent a novel therapeutic approach for improving PH outcomes in offspring exposed to maternal EC.

**Graphical Abstract:**

Pregnant rats were exposed to either EC vapor or standard air from gestation day 12 until 2 days before delivery, with all offspring undergoing PH induction at 6-wo. Offspring exposed to maternal EC presented increased oxidative stress, which in turn affected DNA methylation patterns. The decreased DNA methylation in male offspring led to the activation of autophagy, exacerbating the development of PH. Treatment with ATG5 siRNA inhibited autophagy and alleviated heightened PH in male offspring with maternal EC exposure.

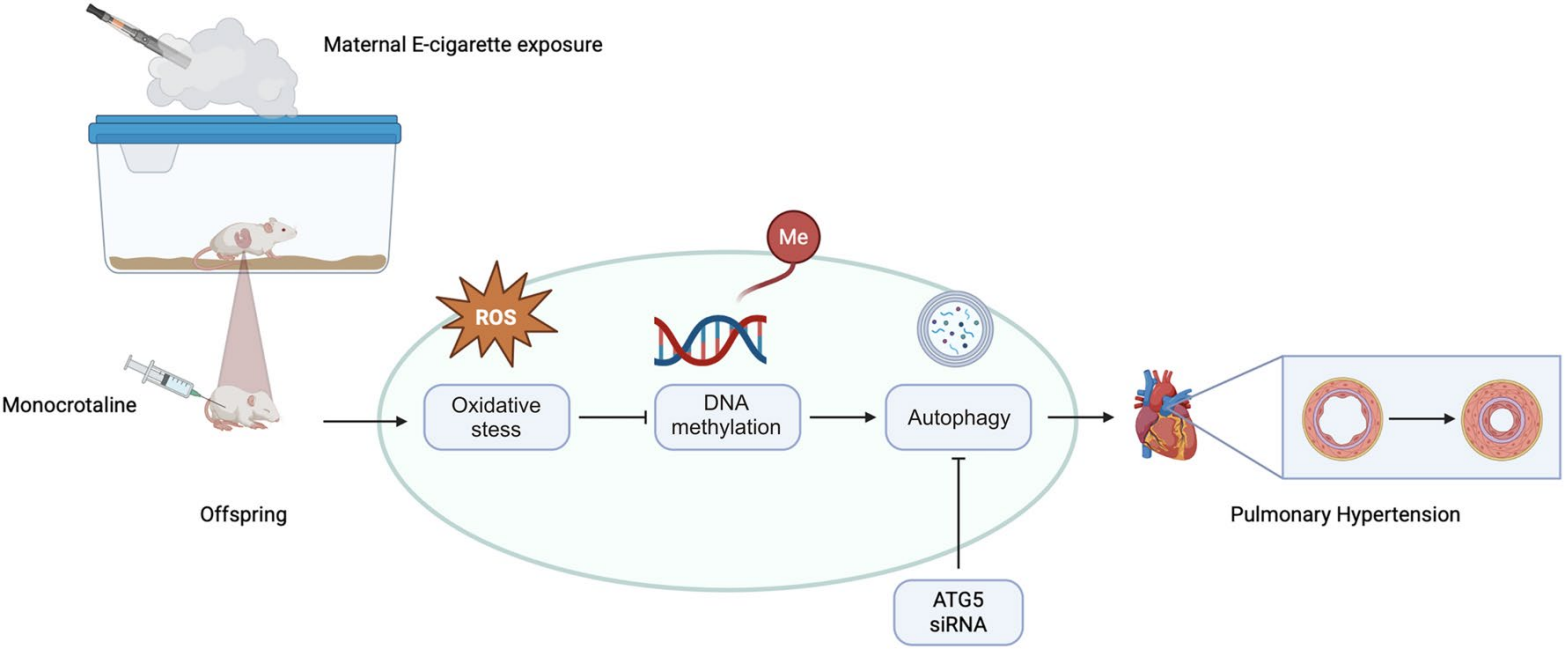

## Introduction

Electronic cigarettes (ECs) are portable battery-powered devices that heat and vaporize an e-liquid solution, enabling users to inhale its contents [1]. Typically, this e-liquid contains nicotine, propylene glycol, glycerin, and various flavoring additives chosen according to personal preferences [2]. Notably, the aerosols produced by ECs differ significantly from those produced by traditional tobacco smoke. Although the levels of lead and chromium are similar in both EC aerosols and traditional smoke, the concentration of nickel is significantly higher in EC aerosols—ranging from 2 to 100 times greater than that in traditional tobacco smoke [3, 4]. Furthermore, nicotine concentrations in traditional tobacco smoke usually range from 10 mg–15 mg per cigarette, whereas ECs deliver nicotine at variable concentrations (0 mg/mL–36 mg/mL), with many European countries capping this amount at 20 mg/mL [5]. Moreover, EC aerosols are characterized by a bimodal particle size distribution with elevated concentrations of nanoparticles, which may contribute to a more pronounced toxicological impact than traditional tobacco smoke does [4]. Numerous studies have highlighted the close association between EC usage and various cardiopulmonary pathologies, leading to increased right ventricle (RV) free wall thickness, heightened inflammation, and oxidative stress, thereby inducing distinct cardiopulmonary remodeling [6, 7].

Pulmonary hypertension (PH) is a life-threatening condition that primarily affects infants, children, and adolescents with severe heart, lung, or systemic disorders. This condition has a 5-year mortality rate of 25%–60% in this population, particularly when associated with congenital heart defects [8, 9]. PH is characterized by the remodeling of pulmonary arteries (PAs) with abnormal extracellular matrix (ECM) deposits, which are closely linked to RV remodeling. Prolonged PH can worsen RV remodeling and potentially lead to right heart failure, a critical factor contributing to mortality [10–12]. Previous research has underscored the role of nicotine in elevating RV systolic pressure and thickening the RV free wall [13]. Additionally, prenatal exposure to ECs has been linked to excessive ECM deposition in the lungs of adult offspring [14]. However, the adverse effects of maternal EC exposure as a contributing risk factor that exacerbates PH in offspring, along with the underlying mechanisms, remain unexplored.

Autophagy, a crucial conserved cellular process, plays a pivotal role in maintaining cellular homeostasis [15]. Recent studies have illuminated the association between autophagic balance and pH. Investigations into the ATG-LC3 system revealed increased autophagy, marked by upregulated LC3B-II, in PA tissues from monocrotaline (MCT)-induced PH rats [16]. Similarly, increased LC3B-II levels have been observed in RV tissues following PH-induced remodeling [17]. The proliferation of pulmonary artery endothelial cells (PAECs) is closely linked to PH progression. PAECs stimulated with MCT exhibited increased proliferation and autophagy, alongside elevated Beclin1 expression. The inhibition of autophagy has shown promise in ameliorating endothelial dysfunction and suppressing abnormal PAEC proliferation in PH [18]. When inhibited, the core component of autophagy, ATG5, has the potential to alleviate excessive proliferation of pulmonary artery smooth muscle cells (PASMCs) induced by platelet-derived growth factor-BB [19, 20]. Despite these findings, the connection between maternal EC exposure and autophagy in PH offspring remains largely unexplored.

Recent studies have highlighted the regulatory role of oxidative stress and alterations in DNA methylation patterns in modulating autophagy [15, 21–23], both of which are biological processes associated with EC exposure [24, 25]. Therefore, this study aimed to investigate the potential association between maternal EC exposure and autophagy in PH offspring. Specifically, we explored the relationship between maternal EC exposure and oxidative stress/DNA methylation in offspring. Furthermore, we sought to identify potential therapeutic targets for PH offspring exposed to maternal EC, aiming to offer novel insights into intervention strategies.

## Materials and Methods

### Animal Preparation

Specific pathogen-free (SPF) pregnant Sprague‒Dawley rats (gestation day 10) were obtained from Beijing Vital River Laboratory Animal Technology Co., Ltd. (China) for the experiments. The animal rooms were maintained at 22°C with 60% relative humidity. All pregnant rats had ad libitum access to fresh food and water. After a 2-day acclimation period in the vivarium, the rats were randomly assigned to two cage groups for the maternal EC exposure study.

Rats (*n* = 12) housed with EC delivery systems were exposed to EC vapor daily from 8:00 a.m. to 8:00 p.m. Each EC puff lasted 4 s with a 30-s interval, amounting to 3 puffs per hour, as described in previous studies [26]. The maternal EC exposure period extended from gestation day 12 to 2 days before the expected delivery date. Rats (*n* = 5) housed in standard cages without EC exposure were monitored over the same timeline as those in the exposed group. All pregnant rats were allowed to give birth, resulting in 17 dams. The offspring were divided into two groups: the air control group and the maternal EC exposure group. The offspring were weaned at 3 weeks of age (3-wo).

To establish an autophagy inhibition model, chloroquine (CQ) (MedChemExpress, USA) was administered intraperitoneally at a dose of 20 mg/kg once a week from 3- to 6-wo in maternal EC-exposed offspring rats, as described in previous studies [27, 28]. To inhibit reactive oxygen species (ROS) generation, maternal EC-exposed offspring were treated with the antioxidant NAC (500 mg/kg/day) (MedChemExpress, USA) in the drinking water from 3- to 6-wo. The rationale for using this dosage and duration of NAC is based on previous studies, which demonstrated its high effectiveness in inhibiting ROS with minimal side effects [29, 30]. Maternal EC-exposed offspring rats infected with adeno-associated virus type 9 (AAV9) containing rat DNMT3B cDNA (AAV9.DNMT3B) (PackGene Biotech, China) via tail-vein injection (1 × 10^11^ vg/100 μL/rat) to achieve DNA hypermethylation at 3-wo. To downregulate ATG5 expression, AAV9.siRNA-ATG5 (PackGene Biotech, China) was administered via tail-vein injection at a dose of 1 × 10^11^ vg/100 μL/rat in maternal EC-exposed offspring rats at week of age following the manufacturer’s instructions. All the rats were sacrificed at 3-wo.

### PH Model and Morphological Measurements

The offspring rats received a single subcutaneous injection of MCT (Yuanye, China) at a dose of 60 mg/kg at 6-wo as described previously [31]. Four-week MCT exposure induces progressive PH. To measure the medial thickness of the PA, offspring rats were sacrificed after 4 weeks of MCT exposure (at 10-wo). PA tissues were collected and fixed in 4% paraformaldehyde for 48 h, embedded in paraffin, sectioned at a thickness of 5 μm and stained with hematoxylin and eosin (HE). To minimize unnecessary injury, PH in offspring rats was evaluated via echocardiography (GE Healthcare, USA) rather than invasive transducer catheterization. Right ventricular systolic pressure (RVSP) was measured to assess the severity of PH, as no evidence of right ventricular outflow tract obstruction or pulmonary stenosis was detected.

### Western Blot Analysis

Total protein was isolated from PA tissues from offspring rats, and these tissues were homogenized in RIPA lysis buffer (Beyotime Biotechnology, China) containing PMSF and a cocktail. The homogenates were subsequently centrifuged at 4°C for 10 min at 14,000× *g*, after which the supernatants were collected. The PA samples with equal amounts of total protein were subjected to SDS‒PAGE and then transferred to PVDF membranes. The membrane was subsequently incubated with specific primary antibodies and secondary antibodies as described previously [29]. Primary antibodies against NADPH oxidases (NOX1, NOX2, NOX4) (Abcam, UK), DNA methyltransferases (DNMT1, DNMT3A, DNMT3B) (Abcam), ATG5 (Thermo Fisher Scientific, USA), Beclin1 (Abcam), LC3B (Abcam) and GAPDH (Aksomics, China) were used in this study.

### Measurement of Reactive Oxygen Species (ROS) Levels

The PA tissues mixed with phosphate-buffered saline (PBS) (pH 7.4) were homogenized on ice and then centrifuged at 4°C for 5 min at 10,000× *g*. The supernatants were collected and incubated with the catalyst and DCFH solution sequentially according to the ROS ELISA kit (Baiyi Biotechnology, China). The fluorescence was determined with a Synergy HT Multi-Mode Microplate Reader (Bio-Tek Instruments, USA) at Ex480 nm/Em530 nm.

### 5‐Methylcytosine (5-mC) DNA ELISA

Total DNA was isolated from PA tissues. 5‐Methylcytosine (5‐mC) levels were quantified with a MethylFlash™ Methylated 5mC DNA Quantification Kit (EpigenTek, USA) according to the manufacturer’s instructions. One hundred nanograms of each total DNA sample was used per reaction. For DNA binding, total DNA samples and negative control/positive control samples were mixed with binding solution and then incubated and washed. Methylated DNA capture was achieved by mixing, followed by incubation with capture antibody, detection antibody and enhancer solution. After mixing and incubation with developer solution and stop solution, the absorbance was read at 450 nm to calculate the percentage of 5-mC in total DNA.

### Statistical Analysis

GraphPad Prism 10.0.2 was used for the statistical analyses. The data are presented as the means ± SEMs. The data were analyzed via *t*-test or two-way ANOVA, where appropriate. In all the cases, *P* values less than 0.05 were considered statistically significant.

## Results

### Effects of Maternal EC Exposure on RV Remodeling in Offspring

To investigate the potential cardiovascular implications of maternal EC exposure, the pregnant rats were exposed to either EC vapor or standard air from gestation day 12 until 2 days before delivery. No significant differences in litter size were detected between the dams in the air-exposed control group and those in the maternal EC exposure group. After birth, all offspring were subjected to MCT treatment to induce a PH model.

**Fig. 1:**
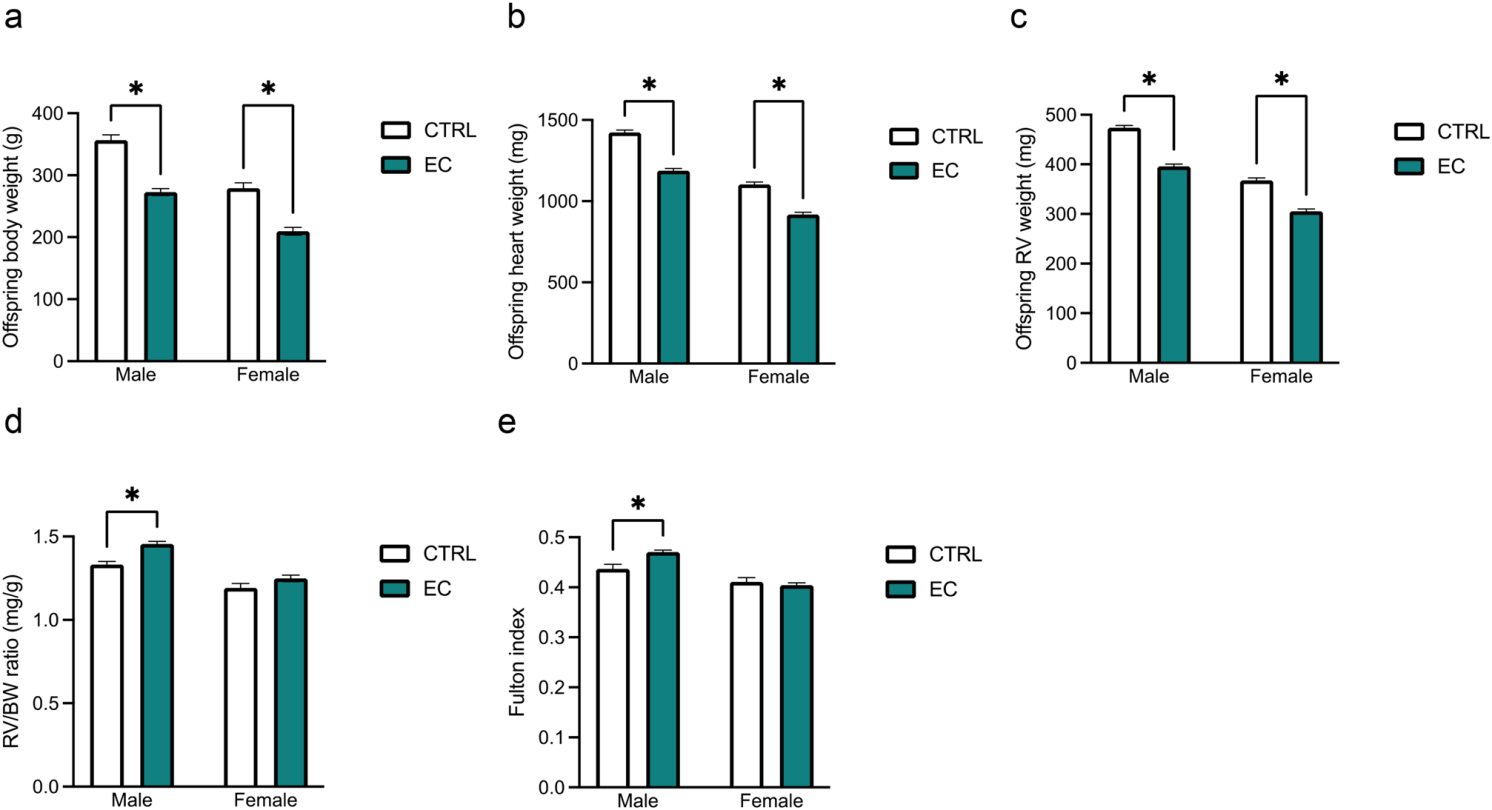
Effect of maternal electronic cigarettes (EC) exposure on right ventricle (RV) remodeling in offspring. Body weight (BW) (**a**), heart weight (HW) (**b**), RV weight (**c**), RV/BW ratio (**d**), and Fulton index (**e**) comparison between offspring from the air control and EC exposure groups at 10 weeks of age (10-wo). The data are presented as the means ± SEMs. ^*^P < 0.05. CTRL: air control group; EC: EC exposure group

Offspring exposed to maternal EC presented significant reductions in body weight (BW) (Fig. 1a), heart weight (HW) (Fig. 1b), and RV weight (Fig. 1c) in both males and females. However, the RV/BW ratio (Fig. 1d), an indicator of RV remodeling, was significantly elevated only in male offspring exposed to maternal EC. Similarly, the Fulton index (Fig. 1e), which is independent of BW, markedly increased exclusively in male offspring subjected to maternal EC exposure.

### Effects of Maternal EC Exposure on PH in Offspring

**Fig. 2:**
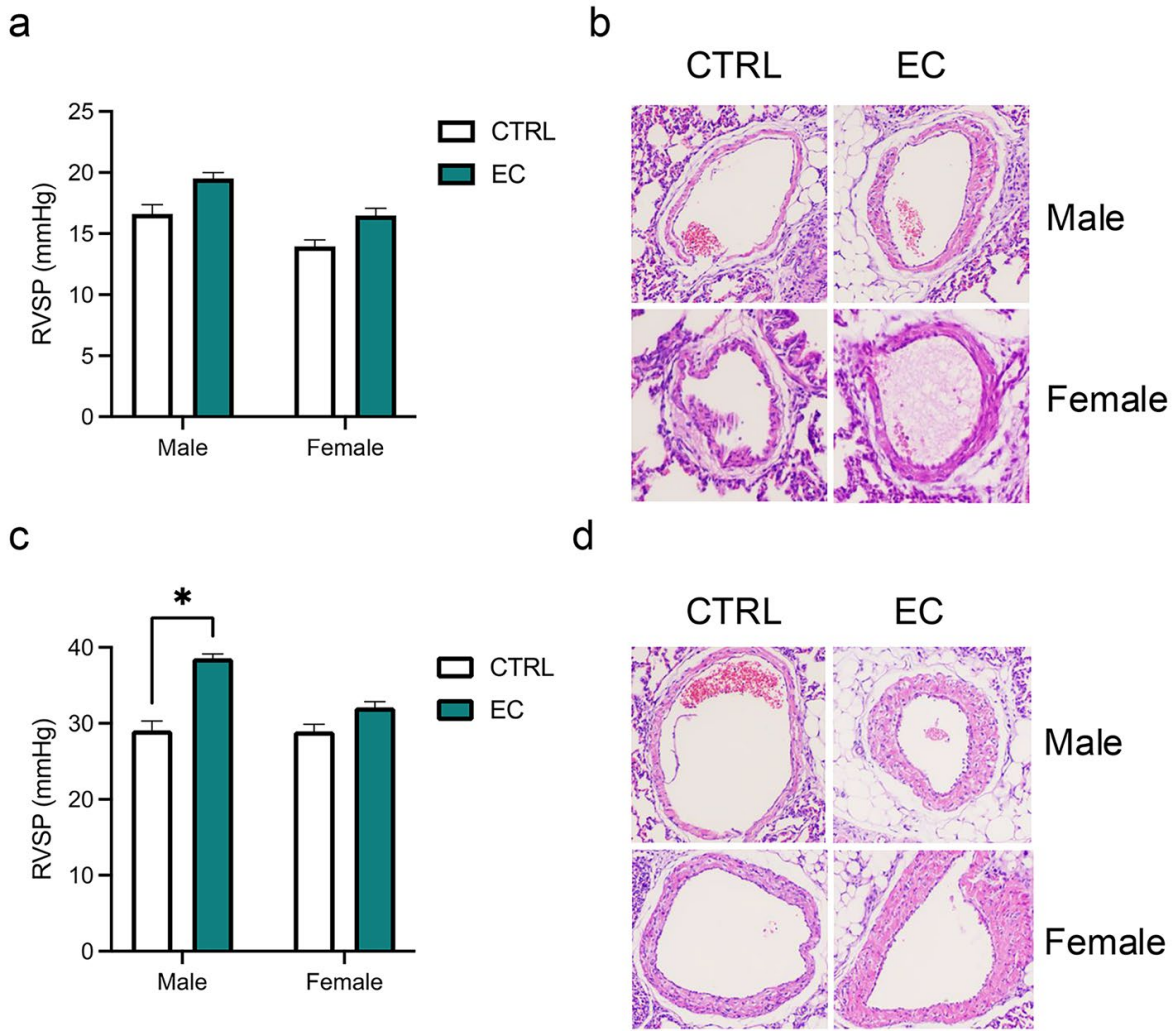
The effect of maternal EC exposure on pulmonary hypertension (PH) development in offspring was evaluated via echocardiography and hematoxylin and eosin (HE) staining. **a, b** comparison of right ventricular systolic pressure (RVSP) and the percentage of medial thickness (%MT) between offspring from the air control (CTRL) and EC exposure groups at 6-wo (original magnification, ×200); **c, d** comparison of RVSP and %MT comparison between offspring from the CTRL and EC exposure groups at 10-wo (original magnification, ×200). The data are presented as the means ± SEMs. ^*^P < 0.05

At baseline (6-wo), no significant differences in RVSP (Fig. 2a) or the percentage of medial thickness (%MT) (Fig. 2b) were detected between male and female offspring from the air control group and those exposed to maternal EC. However, 4 weeks after MCT treatment (10-wo), RVSP was significantly elevated exclusively in male offspring exposed to maternal EC (Fig. 2c). Similarly, the %MT was markedly greater only in male offspring exposed to maternal EC than in their air control counterparts (Fig. 2d).

### Effects of Maternal EC Exposure on Autophagy-Related Protein Expression in PH Offspring

Since no significant differences in PH development were observed in female offspring between the maternal EC exposure group and the air control group, subsequent analyses were conducted exclusively in male offspring.

**Fig. 3:**
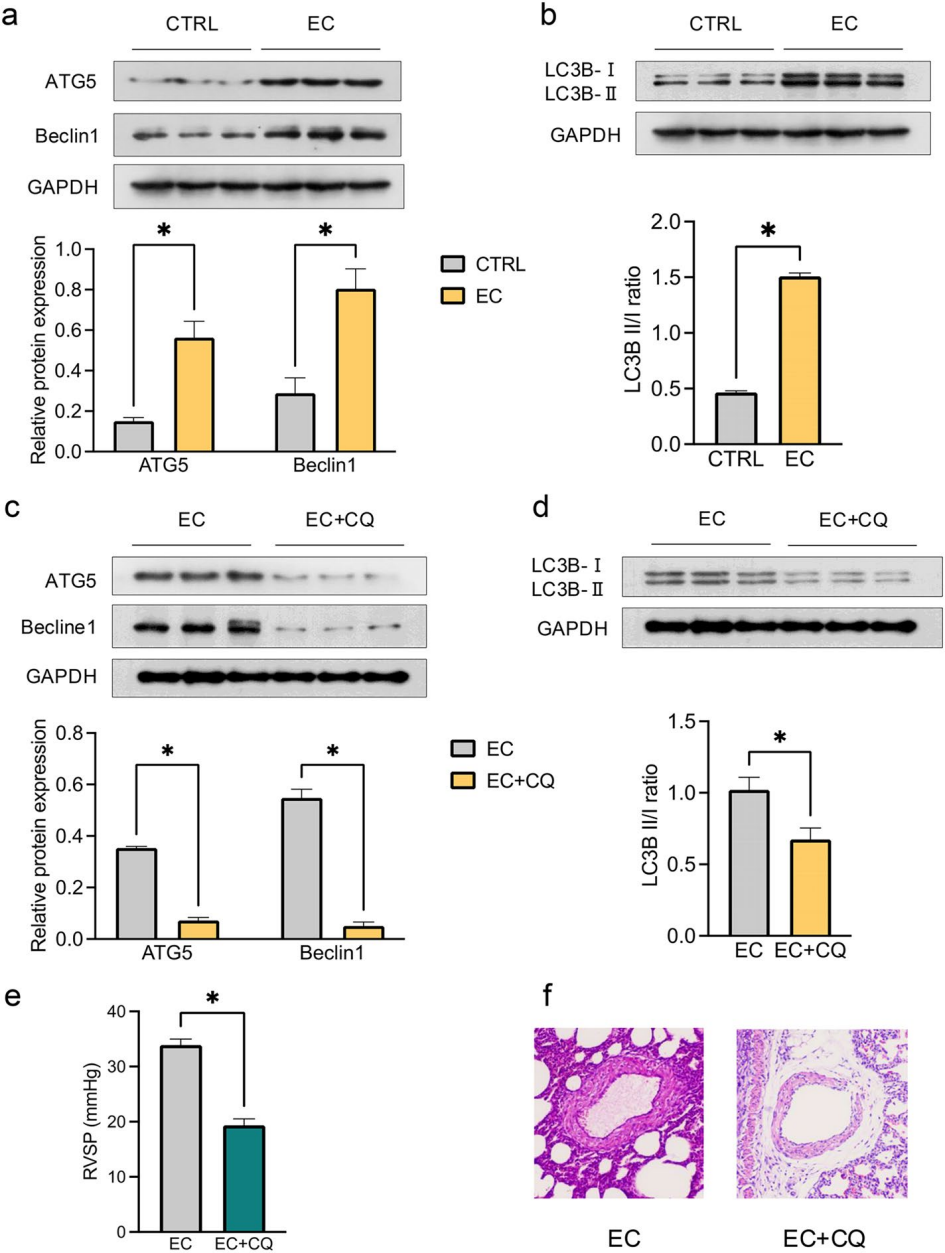
Maternal EC exposure and excessive autophagy in PH offspring. In pulmonary arteries (PA) tissues isolated from male offspring, the relative protein expression of ATG5 and Beclin1 (**a**) and the LC3B II/I ratio (**b**) were evaluated by Western blotting, After treatment with chloroquine (CQ) to inhibit autophagy, the protein expression of ATG5 and Beclin1 (**c**), the LC3B II/I ratio (**d**), the right ventricular systolic pressure (RVSP) (**e**), and the %MT (**f**) were analyzed (original magnification, ×200). The data are presented as the means ± SEMs. ^*^P < 0.05. EC + CQ: EC exposure with autophagy inhibition

Maternal EC exposure significantly increased the expression of autophagy-related proteins, including ATG5 and Beclin1 (Fig. 3a), and increased the LC3B II/I ratio (Fig. 3b) in the PA tissues of offspring. In contrast, autophagy inhibition via CQ treatment reduced the relative expression levels of ATG5 and Beclin1 (Fig. 3c) and decreased the LC3B II/I ratio (Fig. 3d) in offspring exposed to maternal EC.

Additionally, autophagy inhibition alleviated PH induced by maternal EC exposure in offspring, as demonstrated by reductions in RVSP (Fig. 3e) and %MT (Fig. 3f). These results indicate a positive regulatory role of autophagy in the progression of EC exposure-induced PH in offspring.

### Effects of Maternal EC Exposure on DNA Methylation and Its Role in the Regulation of Autophagy in PH Offspring

**Fig. 4:**
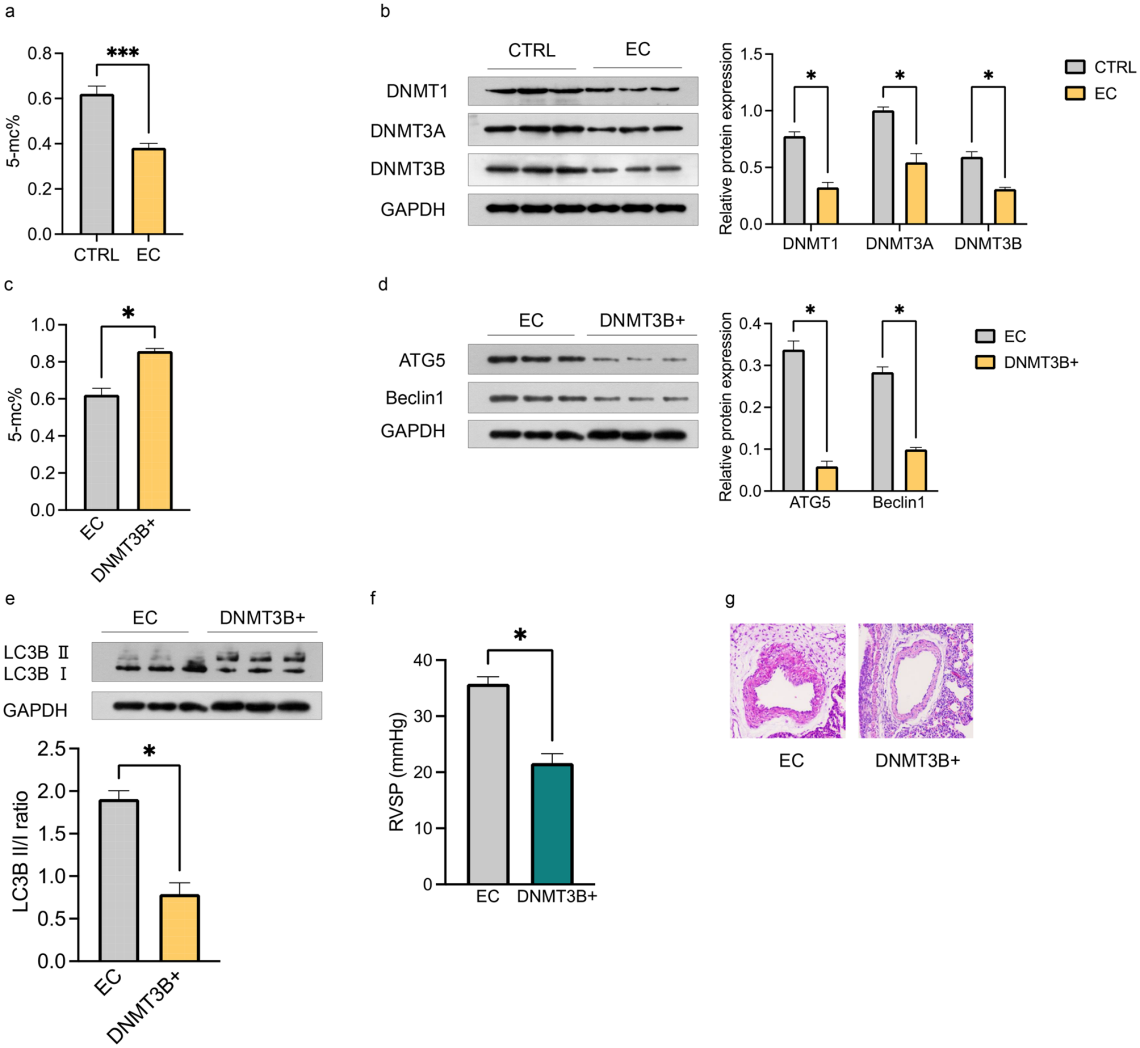
Maternal EC exposure and DNA methylation in PH offspring. **a** Global DNA methylation levels in PA tissues isolated from male offspring; **b** relative protein expression levels of DNMT1, DNMT3A, and DNMT3B determined by Western blotting in PA tissues isolated from male offspring; **c** global DNA methylation levels following hypermethylation treatment; **d, e** autophagic protein expression of ATG5 and Beclin1, as well as the LC3B II/I ratio following hypermethylation treatment; **f, g** comparison of RVSP and %MT between offspring from the non-hypermethylation and hypermethylation groups (original magnification, ×200). The data are presented as the means ± SEMs. ^*^P < 0.05; ^***^P < 0.001. 5-mc: 5‐methylcytosine; DNMT3B+: with AAV9.DNMT3B treatment

To explore the upstream mechanisms underlying the increased autophagy levels observed in PH offspring due to maternal EC exposure, we investigated the role of DNA methylation. Compared with those from the control group, offspring exposed to maternal EC presented significantly lower global DNA methylation levels (Fig. 4a). This decrease in DNA methylation was accompanied by reduced protein expression of DNMT1, DNMT3A, and DNMT3B (Fig. 4b).

To further examine the functional role of DNA methylation, PH offspring were treated with AAV9.DNMT3B to induce DNA hypermethylation. This treatment effectively restored global DNA methylation levels (Fig. 4c). In offspring from the maternal EC exposure group, hypermethylation resulted in decreased protein expression of ATG5 and Beclin1 (Fig. 4d) and a reduced LC3B II/I ratio (Fig. 4e).

Importantly, DNA hypermethylation ameliorated the PH phenotype in maternal EC-exposed offspring, as demonstrated by reductions in RVSP (Fig. 4f) and %MT (Fig. 4f). These findings suggest that DNA methylation acts as a negative regulator of EC exposure-induced PH in offspring by modulating autophagy pathways.

### Effects of Maternal EC Exposure on Oxidative Stress and Its Role in Regulation of Autophagy in PH Offspring

**Fig. 5:**
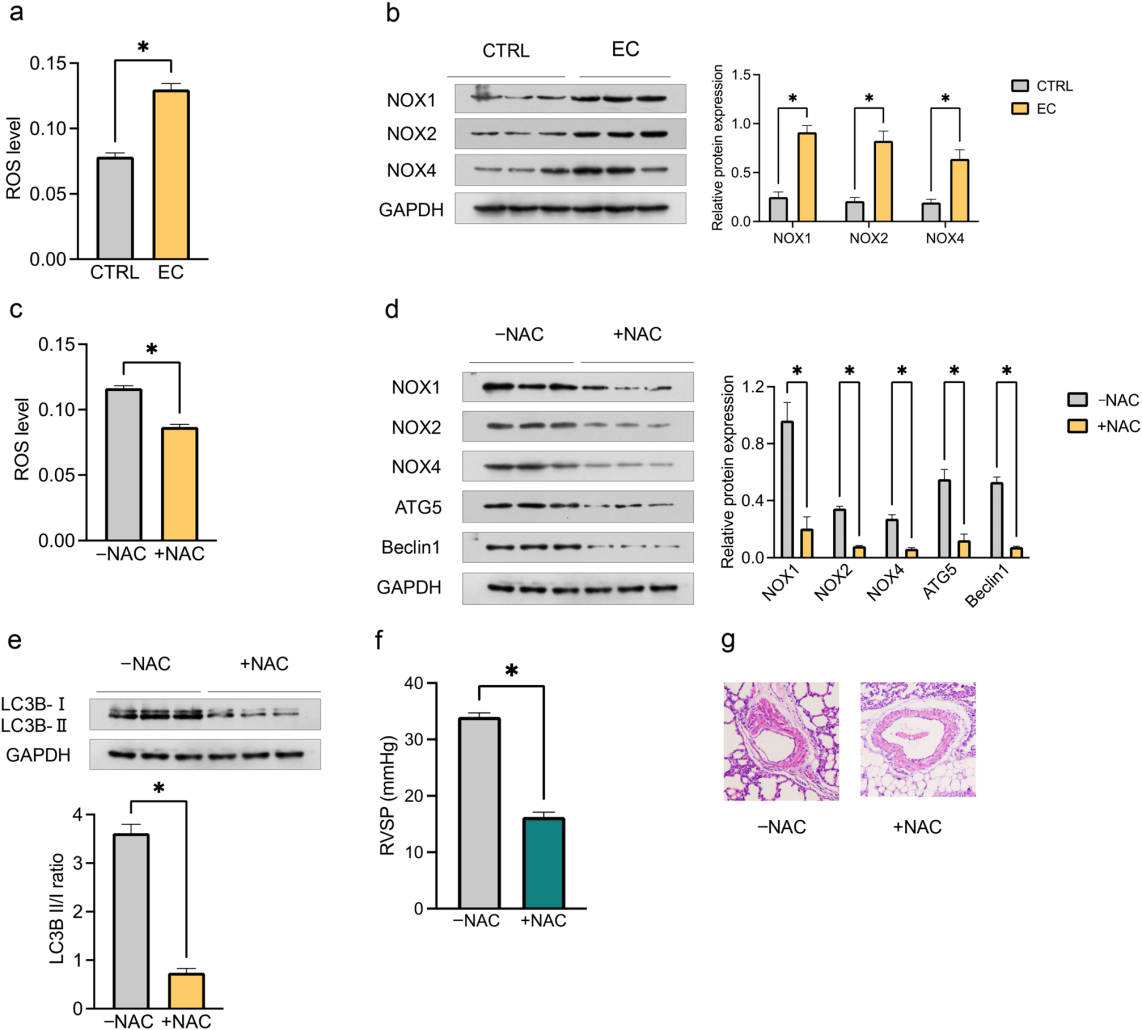
Maternal EC exposure and oxidative stress in PH offspring. **a** The ROS levels in PA tissues isolated from male offspring; **b** the relative protein expression levels of NOX1, NOX2, and NOX4 determined via Western blotting; **c–e** the ROS level (**c**), relative protein expression of NOX1, NOX2, and NOX4, autophagic protein expression of ATG5 and Beclin1 (**d**), and the LC3B II/I ratio (**e**) following N-acetylcysteine (NAC) treatment; **f, g** comparison of RVSP and %MT between offspring from the −NAC and +NAC groups (original magnification, ×200). The data are presented as the means ± SEMs. *P < 0.05. −NAC: without NAC treatment; +NAC: with NAC treatment

Maternal EC exposure resulted in a significant increase in ROS levels (Fig. 5a) in PA tissues, coupled with increased protein expression of NOX1, NOX2 and NOX4 (Fig. 5b) in offspring. Introducing the antioxidant NAC through drinking water effectively suppressed ROS levels (Fig. 5c) and reduced the protein expression of NOX1, NOX2 and NOX4 (Fig. 5d) in offspring exposed to maternal EC.

Furthermore, the presence of NAC coincided with decreased relative protein expression of ATG5 and Beclin1 (Fig. 5d) and a reduction in the LC3B II/I ratio (Fig. 5e) in offspring exposed to maternal EC. Similarly, NAC administration mitigated the increased RVSP (Fig. 5f) and increased %MT (Fig. 5g) induced by maternal EC exposure in offspring. These observations suggest that the impact of oxidative stress on EC exposure-induced PH in offspring may operate through the regulation of autophagy.

### Oxidative Stress Induces Autophagy by Inhibiting DNA Methylation in PH Offspring Exposed to Maternal EC

**Fig. 6:**
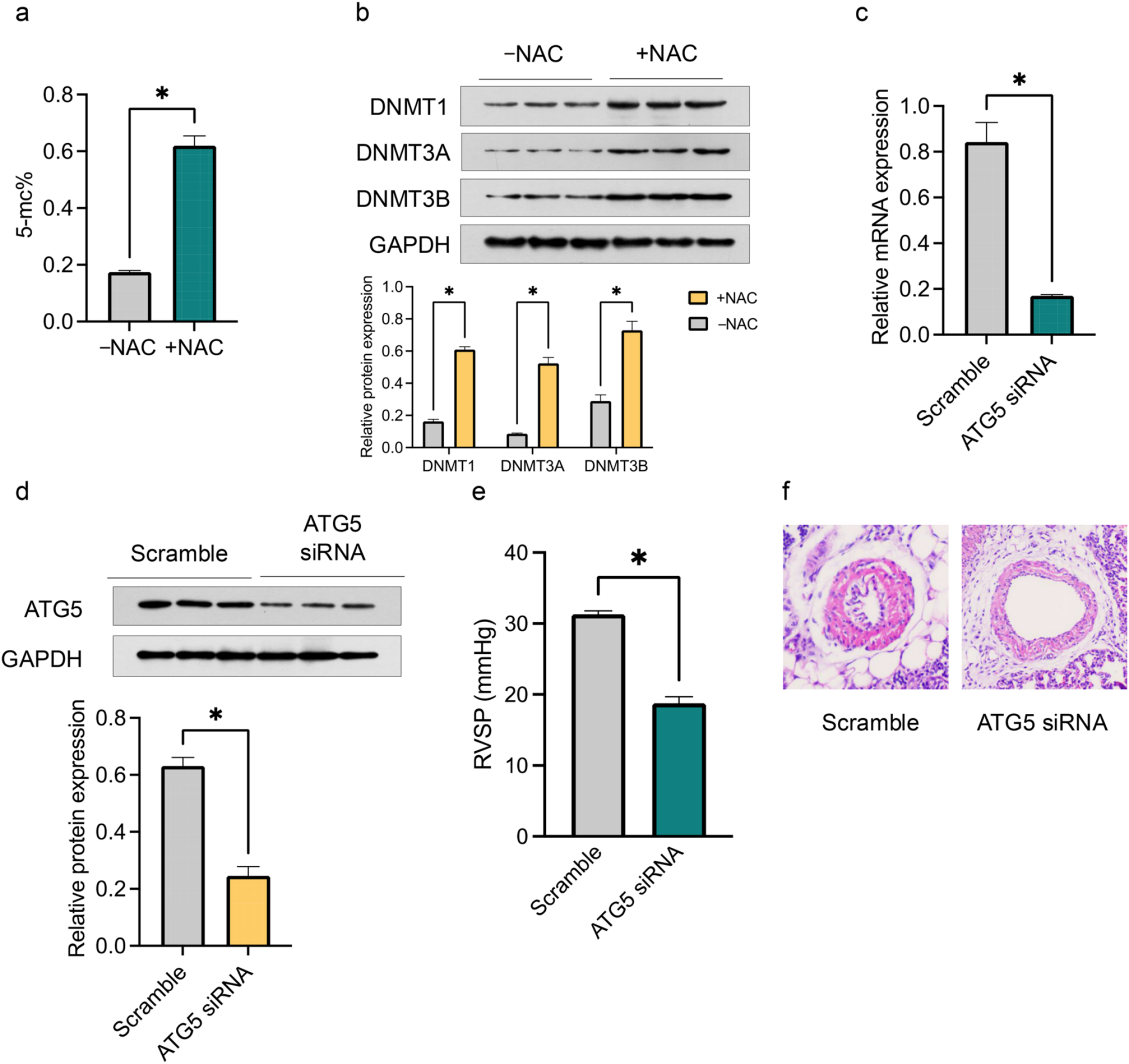
Oxidative stress/DNA methylation/autophagy axis in PH development in offspring exposed to maternal EC. **a** Global DNA methylation levels in PA tissues isolated from male offspring; **b** the relative protein expression levels of DNMT1, DNMT3A, and DNMT3B determined via Western blotting; **c** ATG5 mRNA expression via qRT‒PCR; **d** ATG5 protein expression analyzed via Western blotting; **e** RVSP measured via echocardiography; **f** %MT evaluated via HE staining (original magnification, ×200). The data are presented as the means ± SEMs. *P < 0.05. ATG5 siRNA: with AAV9.siRNA-ATG5 treatment

Our previous findings demonstrated that maternal EC exposure induces oxidative stress and DNA hypomethylation, which exacerbates PH by promoting autophagy in offspring. These effects were reversed by the antioxidant NAC and by DNA hypermethylation. To further explore the relationship between oxidative stress and DNA methylation, a series of experiments were conducted. NAC administration restored global DNA methylation levels (Fig. 6a) and increased the expression of the DNMT1, DNMT3A, and DNMT3B proteins (Fig. 6b) in offspring exposed to maternal EC. These findings, combined with previous results showing that DNA hypermethylation mitigates oxidative stress-induced autophagy and PH (Fig. 4d–g), indicate the involvement of an oxidative stress/DNA methylation/autophagy axis in the development of PH in offspring exposed to maternal EC.

Given the critical role of ATG5 in autophagy, we used ATG5 siRNA to specifically target ATG5 expression and modulate autophagy in offspring. ATG5 siRNA effectively reversed the elevated ATG5 mRNA levels induced by maternal EC exposure (Fig. 6c), resulting in a significant reduction in ATG5 protein expression (Fig. 6d). Consequently, the abnormal RVSP (Fig. 6e) and %MT (Fig. 6f) observed in offspring were alleviated in the ATG5 siRNA-treated group compared with those in the scramble control group.

These findings highlight the role of oxidative stress in inducing autophagy via DNA methylation inhibition and suggest that ATG5 could serve as a promising therapeutic target for regulating autophagy in PH offspring resulting from maternal EC exposure.

## Discussion

Smoking remains a primary preventable cause of mortality and morbidity worldwide. Its impact during pregnancy is well documented and is associated with increased risks of miscarriage, fetal growth limitations, preterm birth, and congenital anomalies in offspring, manifesting even into later stages of childhood [32, 33]. EC has been marketed as a safer alternative to traditional tobacco smoke, touted as a potentially less risky aid for smoking cessation [34]. Nonetheless, preceding studies have revealed the outcomes of in utero EC exposure, which disrupts various physiological functions in offspring, including the cerebrovascular [35], pulmonary [36], hepatic [37], and renal systems [38]. Notably, fetal exposure to ECs has been shown to reduce BW and brain weight while increasing the brain-to-BW ratio in neonates [39]. Similarly, our present findings indicate that maternal EC exposure leads to reduced BW, HW and RV weights, suggesting a potential link to offspring growth restriction. Moreover, the elevated RV/BW ratio and Fulton index in offspring from EC-exposed mothers point toward an asymmetric alteration in right heart development, potentially contributing to RV remodeling. However, these findings beg the following question: what further anatomical anomalies might arise?

Animal studies have associated EC usage with increased RV systolic pressure and RV free wall thickness, culminating in RV remodeling and PH [13, 40]. Interestingly, chronic inhaled nicotine-induced PH does not exhibit a sex-based disparity, with ovarian hormones seemingly not mediating cardiopulmonary protective mechanisms in this context [40]. PA remodeling plays a pivotal role in the development and progression of PH [41]. Our study reinforces this finding by revealing how maternal EC exposure intensifies the severity and advancement of PH in offspring. Before PH modeling, PA remodeling assessment via HE staining revealed no difference between male offspring from the air control group and those exposed to maternal ECs. However, after PH induction, the medial PA wall in male offspring from EC-exposed mothers was thicker than that in the air control group. Notably, we acknowledge a limitation due to the lack of data on female offspring. A recent review suggested that while females are more susceptible to PH, they tend to have better outcomes than male PH patients are [42]. In our previous study, we highlighted the potential protective role of estrogen in cardiovascular health in female offspring, particularly in response to adverse intrauterine environments later in life. These estrogen-related protective mechanisms involve the modulation of the renin‒angiotensin system, antioxidant effects and the regulation of nitric oxide-mediated signaling [43]. Consequently, we focused on male offspring for data collection. However, this does not preclude the potential impact of maternal EC exposure on PH in female offspring. Future research focused on female offspring could provide valuable insights into the sex-dependent effects of maternal EC exposure.

Autophagy, an important cellular response mechanism to adverse conditions, balances “cell death” with normal cell functions [44]. Studies have suggested that increased autophagy can suppress pulmonary vascular cell proliferation, potentially ameliorating the severity of PH [44, 45]. However, conflicting reports indicate that inhibiting certain pathways regulating autophagy might have a converse effect on PH [20]. The reason for this discrepancy may be the result of disruption of the balance of autophagy. Our study revealed increased protein expression of ATG5 and Beclin 1 and an increased LC3B II/I ratio in male offspring following maternal EC exposure, suggesting an increase in autophagy. CQ is a well-known autophagy inhibitor that suppresses lysosomal acidification [46]. Administering CQ to male offspring mitigated PA remodeling post-PH insult, indicating a role for excessive autophagy in exacerbating PH caused by maternal EC exposure. This prompted us to investigate the following questions: what factors regulate autophagy upstream?

Increased oxidative stress is a fundamental mechanism underlying smoking-related cardiovascular diseases. A review by Shahandeh et al. published in Heart demonstrated that compared with nonsmokers, EC users exhibit elevated oxidative stress levels [47]. Recent studies have also revealed oxidative damage following EC exposure in both mouse models and human cells in laboratory settings [48–50]. Similarly, our study revealed increased ROS production and upregulated protein expression of NOX1, NOX2, and NOX4 in male offspring from EC-exposed mothers. Elevated oxidative stress implicates ROS in the pathophysiological changes observed in PH offspring resulting from maternal EC exposure. ROS can influence cell apoptosis, proliferation, and differentiation via the regulation of autophagy, a phenomenon observed in pulmonary disorders such as pulmonary fibrosis and PH [19, 51]. Administering the antioxidant NAC in our study not only reduced ROS levels following maternal EC exposure in male offspring but also reversed the overexpression of autophagy-related proteins, subsequently increasing PH severity. These findings underscore the role of the interplay between ROS and autophagy as a significant molecular mechanism underlying the worsening of PH caused by maternal EC exposure.

DNA methylation, an epigenetic mechanism that governs gene expression, has been linked to cardiovascular diseases [52, 53] and PH [54]. Adverse intrauterine conditions have been shown to alter offspring DNA methylation, impacting downstream pathways [29, 55]. Our study revealed reduced global DNA methylation levels in male offspring from EC-exposed mothers, accompanied by suppressed protein expression of DNMT1, DNMT3A, and DNMT3B. Introducing AAV9.DNMT3B restored global DNA methylation levels in these male offspring, concurrently reducing autophagy-related protein expression. Similar associations between DNA methylation and autophagy have been reported in diverse conditions, such as lung cancer [56], pulmonary fibrosis [57] and diabetic cardiomyopathy [58]. These findings implicate DNA methylation in exacerbating PH due to maternal EC exposure, potentially through the upregulation of autophagy.

In a population-based study on epigenetic traditional tobacco smoking indicators and oxidative stress, alterations in smoking-related DNA methylation patterns were closely correlated with smoking-induced oxidative stress. The DNA methylation changes at the identified CpG sites could serve as prognostic epigenetic markers of smoking-induced oxidative stress, although the underlying mechanisms require further investigation [59]. Research on the relationship between oxidative stress and DNA methylation has focused primarily on conditions such as metabolic syndrome and Alzheimer’s disease [47, 60, 61]. In the progression of renal fibrosis, ROS can promote hypermethylation of the NDRG2 promoter [62], which is consistent with findings in diabetic retinopathy, where inhibiting DNMT1 was shown to reduce ROS levels [63]. However, in a study on carcinogenesis, ROS were found to induce the conversion of guanine to 8-hydroxy-2′-deoxyguanosine and 5-methylcytosine to 5-hydroxymethylcytosine, leading to global DNA hypomethylation [64]. Furthermore, in a study on developing mouse embryos, NAC, an antioxidant, was shown to increase the levels of all DNMTs and global methylation, influencing DNA methylation patterns [65]. In the present study, NAC treatment, which reduced ROS levels, led to increased DNMTs expression and induced DNA hypermethylation in male offspring exposed to maternal EC. This interplay between ROS and DNA methylation suggests an epigenetic mechanism underlying PH in offspring due to maternal EC exposure, which warrants further investigation into specific promoter methylation patterns.

ATG5, a critical autophagy-related protein that regulates the autophagic process [66], has garnered increasing attention in the context of perinatal nicotine exposure [67, 68]. Our study aligns with previous reports, which revealed significantly greater ATG5 mRNA and protein expression in male offspring following maternal EC exposure. Silencing ATG5 alleviated heightened PH in these offspring, suggesting a potential avenue for PH therapy despite the oxidative stress and DNA methylation anomalies induced by maternal EC exposure. However, understanding whether the regulation of the methylation pattern of the ATG5 promoter occurs through the oxidative stress/DNA methylation mechanism requires more comprehensive research.

## Conclusions

Considering the widespread use of ECs, including among pregnant women, it is imperative to explore the potential adverse effects of EC on offspring. PH poses a significant life-threatening challenge, especially since untreated patients face the risk of mortality due to right heart failure, with no current cure available. While the role of ECs in the pathophysiology of PH is gaining attention, research on whether maternal EC exposure impacts PH development in offspring is limited. Our study represents a pioneering investigation, providing initial evidence that maternal EC exposure amplifies the severity and progression of PH in male offspring. Our data underscore that PH development in male offspring exposed to maternal ECs stems from a cascade involving oxidative stress/DNA methylation-induced aberrant activation of autophagy. Furthermore, the silencing of ATG5 reversed the impact of oxidative stress/DNA methylation on autophagy and notably ameliorated PH in male offspring resulting from maternal EC exposure. These findings reveal a potential avenue for intervention: the potential influence of ATG5 on autophagy-based therapy, which is promising for treating or preventing PA remodeling in PH secondary to maternal EC exposure. As the understanding of these mechanisms evolves, exploring therapeutic approaches targeting autophagy pathways, possibly through ATG5 modulation, holds potential for mitigating the deleterious effects of maternal EC exposure on PH development in offspring. These findings underscore the critical need for further research to delineate these mechanisms and ascertain their clinical implications for potential therapeutic interventions.

## Data Availability

The datasets used and/or analyzed during the current study are available from the corresponding author upon reasonable request.
